# Efficacy and Safety of Tai Chi for Parkinson's Disease: A Systematic Review and Meta-Analysis of Randomized Controlled Trials

**DOI:** 10.1371/journal.pone.0099377

**Published:** 2014-06-13

**Authors:** Xiaojia Ni, Shaonan Liu, Fuchang Lu, Xiaogeng Shi, Xinfeng Guo

**Affiliations:** 1 Research and Development Department of New Drugs, Guangdong Provincial Hospital of Chinese Medicine, Guangzhou, Guangdong Province, China; 2 The Second College of Clinical Medicine, Guangzhou University of Chinese Medicine, Guangzhou, Guangdong Province, China; 3 Key Unit of Methodology in Clinical Research, Guangdong Provincial Hospital of Chinese Medicine, Guangzhou, Guangdong Province, China; 4 Department of Neurology, Guangdong Provincial Hospital of Chinese Medicine, Guangzhou, Guangdong Province, China; Cardiff University, United Kingdom

## Abstract

**Background and Objective:**

In Parkinson's disease (PD), wearing off and side effects of long-term medication and complications pose challenges for neurologists. Although Tai Chi is beneficial for many illnesses, its efficacy for PD remains uncertain. The purpose of this review was to evaluate the efficacy and safety of Tai Chi for PD.

**Methods:**

Randomized controlled trials (RCTs) of Tai Chi for PD were electronically searched by the end of December 2013 and identified by two independent reviewers. The tool from the Cochrane Handbook 5.1 was used to assess the risk of bias. A standard meta-analysis was performed using RevMan 5.2 software.

**Results:**

Ten trials with PD of mild-to-moderate severity were included in the review, and nine trials (n = 409) were included in the meta-analysis. The risk of bias was generally high in the blinding of participants and personnel. Improvements in the Unified Parkinson's Disease Rating Scale Part III (mean difference (MD) −4.34, 95% confidence interval (CI) −6.67–−2.01), Berg Balance Scale (MD: 4.25, 95% CI: 2.83–5.66), functional reach test (MD: 3.89, 95% CI: 1.73–6.04), Timed Up and Go test (MD: −0.75, 95% CI: −1.30–−0.21), stride length (standardized MD: 0.56, 95% CI: 0.03–1.09), health-related quality of life (standardized MD: −1.10, 95% CI: −1.81–−0.39) and reduction of falls were greater after interventions with Tai Chi plus medication. Satisfaction and safety were high. Intervention with Tai Chi alone was more effective for only a few balance and mobility outcomes.

**Conclusions:**

Tai Chi performed with medication resulted in promising gains in mobility and balance, and it was safe and popular among PD patients at an early stage of the disease. This provides a new evidence for PD management. More RCTs with larger sample size that carefully address blinding and prudently select outcomes are needed. PROSPERO registration number CRD42013004989.

## Introduction

Parkinson's disease (PD) is a progressive neurodegenerative disorder characterized by static tremor, rigidity, bradykinesia and postural disturbance [1] with a crude incidence rate of 4.5 to 19 per 100,000 people globally [2]. It afflicts 1.7% of the Chinese population >65 years old, a similar prevalence to that reported in developed countries [3]. The management of PD is complex and individualized. Levodopa replacement therapy and various other drug combinations achieve gains in the early stage of the disease, especially in motor impairments [4]. However, with the progress of the disease and wearing off of the medication, long-term side effect involving symptom fluctuation, dyskinesia and psychiatric comorbidity [5] is a great challenge for neurologists. Infusion therapy [6] and functional surgery such as deep brain stimulation [7] have emerged to address the unsolved problems induced by classical medications, yet their efficacy and safety require further validation. In addition to the major motor symptoms caused by PD, patients experience complications including insomnia, excessive daytime sleepiness, autonomic dysfunction, dementia and depression that are typically tackled by combined drug therapy with unknown risks [8].

Increasing evidence is being found that physiotherapy can benefit PD patients [9]. Tai Chi is a balance-based exercise guided by the yin-yang theory of traditional Chinese medicine that combines deep-breath relaxation and slow and gentle movements with awareness [10]. Tai Chi has been reported to reduce the incidence of falls in an elderly population [11], decrease blood pressure [12], improve lung [13] and cardiac [14] functions, alleviate menopausal osteoporosis [15], relieve psychological dysfunction [16], insomnia [17] and low back pain [18], rehabilitate post-stroke syndromes [19], and slow the progression of rheumatic illness [20], [21] and dementia [22]. In light of these gains, Tai Chi shows potential to help both the motor dysfunction and non-motor complications of PD. However, the clinical benefit of Tai Chi in PD remains uncertain due to different study designs, small sample size and inconsistent methodological quality of the published clinical studies. Some trials even pose contradictory results for the same outcome [23].

A systematic review aims to minimize bias and providing more reliable findings by using explicit and systematic method [24], [25]. Also, a meta-analysis adds to the review can increase power of testing, improve precision of estimated effect, settle controversies and answer clinical question not posed by the individual studies [26]. To identity whether Tai Chi safely benefits PD patients, we performed a systematic review and meta-analysis of randomized controlled trials (RCTs) of Tai Chi for PD.

## Methods

### 1. Protocol and registration

The study was prospectively registered and the protocol was specified in advance and documented in the PROSPERO database with the number CRD42013004989.

### 2. Eligibility

Eligible studies were RCTs without restriction of publication language, date or status. Participants of any age, gender and ethnic group were clinically diagnosed as PD in any stage. Tai Chi was practiced alone or in combination with conventional medication, compared to other exercise with or without conventional medication, medication alone, placebo, or no intervention. Intervention duration and length of follow up were not restricted.

The primary outcome was the global scores of Unified Parkinson's Disease Rating Scale Part III (UPDRS III). The UPDRS III is a 14-item instrument that integrates all aspects of motor symptoms caused by PD, and the score ranges from 0 to 56, with lower values indicating less motor disability [27]. Secondary outcomes were: 1) balance and mobility function assessed using the Berg Balance Scale (BBS), the functional reach test (FRT) or the Timed Up and Go test (TUG); 2) health-related quality of life (HRQOL) assessed using the Parkinson's Disease Questionairre-39 (PDQ-39) or the PDQ-39 Summary Index (PDQ-39SI); 3) gait, including velocity and stride length; 4) falls; 5) post-program survey (PPS); and 6) adverse events (AE). The studies finally included should have at least one of these outcomes.

### 3. Information source and search strategy

A systematic search, as recommend by the PRISMA statement [28], was performed in online databases, including PubMed (from 1966), Embase (from 1985), the Cochrane Library, the Chinese Biomedical Database (from 1979), the China National Knowledge Infrastructure (from 1915), VIP Journal Integration Platform (from 1989), Wanfang Med Online (from 1982), and the Japan Medical Abstracts Society using the following search terms: Tai Chi, Tai Ji, T'ai Chi, Taijiquan, Parkinson disease, Parkinson's disease, Primary Parkinsonism and Paralysis Agitans. The websites of ClinicalTrials.gov and the Chinese Clinical Trial Registry were also searched to identify unpublished clinical trials. In addition, a search was performed in Google Scholar to find reports from other sources. The final search was performed on December 31, 2013. The search strategy is specified in the appendix.

### 4. Study selection and data collection

Two authors (Xiaojia Ni and Fuchang Lu) independently identified and selected studies in a standardized manner. The primary search was carried out in all information sources and was followed by duplication screening. Abstracts and full texts were then reviewed for eligibility criteria. The decision on which studies to include in qualitative and quantitative synthesis was made according to the type of outcome data reported. Disagreement was resolved by a third author (Shaonan Liu).

Two authors (Xiaojia Ni and Fuchang Lu) independently extracted the following information from the included trials and entered it into an Excel spreadsheet: (1) study design and number of patients; (2) baseline patient characteristics (age, gender, disease stage or severity and disease history); (3) intervention and control (type, dose, duration, frequency and any medication); (4) outcomes; (5) length and frequency of follow up; and (6) AE. Duplicate publications were carefully detected and incomplete information was dealt with by contacting the investigators for further data. Disagreements were resolved by discussion or by consulting a third author (Shaonan Liu).

### 5. Risk of bias assessment

Two authors (Xiaojia Ni and Shaonan Liu) independently assessed the risk of bias in individual studies using the Cochrane Collaboration tool [29]. When necessary, information contained in the published articles was supplemented by the published protocol, registration records or by contacting the corresponding authors. Any discrepancy was resolved by another author (Xinfeng Guo). The assessment of blinding was made based on different groups of outcomes, including: 1) clinician-reported outcomes (UPDRS III, BBS, FRT, TUG, gait velocity and stride length), 2) subjective patient-reported outcomes (PDQ-39, PDQ-39SI and PPS,), and 3) objective patient-reported outcome (falls). Seven aspects were evaluated, as follows: (1) random sequence generation, (2) allocation concealment, (3) blinding of participants and personnel (healthcare providers), (4) blinding of outcome assessment, (5) incomplete outcome data, (6) selective reporting and (7) other bias. Judgments were categorized as ‘low risk of bias’, ‘high risk of bias’, or ‘unclear risk of bias’. According to our knowledge of Tai Chi and experience of clinical research, other bias was assessed and judgment was made as ‘high risk of bias’ if control group lacking validation, unreliable randomization, or insufficient sample size occurred.

### 6. Data synthesis and analysis

Dichotomous data were presented as relative risk (RR) with 95% confidence interval (CI) and continuous outcomes were presented as mean difference (MD) between intervention and control groups with 95% CI. HRQOL and gait outcomes are presented as standardized MD (SMD) because the scales or units were not consistent across studies.

In studies with multiple groups, the ‘shared’ group was evenly divided into two or more groups with smaller sample size, and two or more (reasonably independent) comparisons were performed. For dichotomous outcomes, both the number of events and the total number of patients were divided. For continuous outcomes, only the total number of participants was divided and the mean and standard deviation was left unchanged.

Review Manager software (RevMan, Version 5.2, Copenhagen: The Nordic Cochrane Centre, The Cochrane Collaboration, 2012) was used for data analysis. Heterogeneity between trials was detected using a chi-square test. For studies with good homogeneity (P>0.10, I_2_<40%), a fixed model was used for the meta-analysis. For all other studies, a random model was used and particular care was taken when interpreting the results. Subgroup analysis was used to compare the effect of Tai Chi with medications to medications alone, and the effect of Tai Chi alone to no intervention. Publication bias was assessed by funnel plot analysis if the group included more than ten trials.

## Results

### 1. Study identification

**Figure 1:**
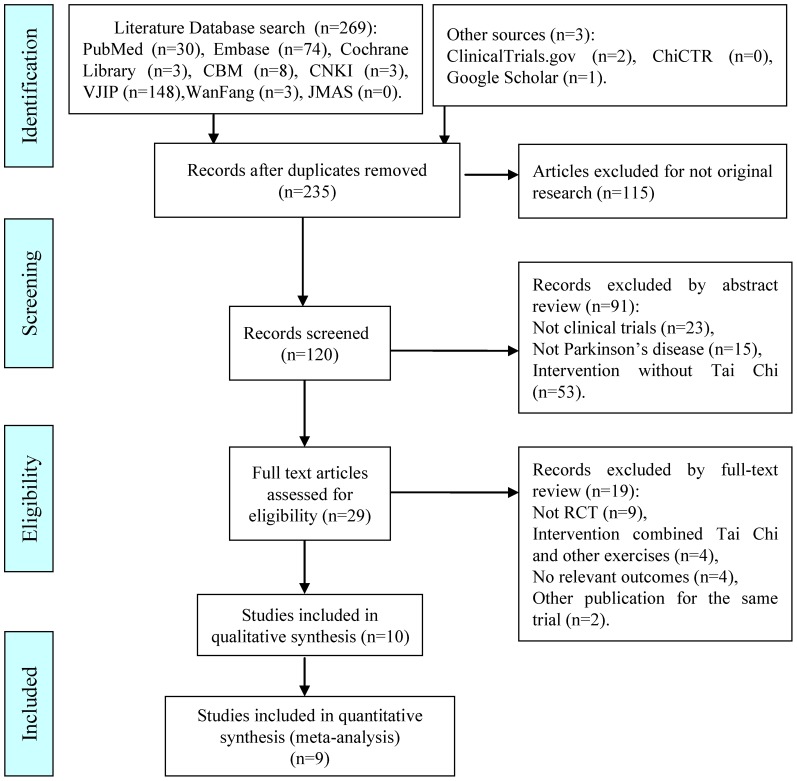
Flow diagram of study selection and identification. The total number of articles and studies differs because one article reported two independent trials and two articles reported different outcomes for the same trial.

A total of ten articles [30]–[39] were included in the review. One article [33] reported two different RCTs and two articles [32], [34] reported different outcomes for the same trial. Eight articles [30]–[35], [38]–[39] were published in English and two [36], [37] were published in Chinese. The flow diagram of search and identification is shown in Figure 1.

### 2. Study characteristics

All trials included were randomized, parallel, controlled clinical trials with blinded assessment. Only two studies [32], [34], [36] were conducted in multiple centers. A total of 470 patients with PD of mild-to-moderate severity (evaluated using Hoehn and Yahr stage) were studied in the review. Seven trials [30]–[32], [34], [36]–[39] used stable medications (e.g., levodopa) as part of the basic treatment and the others [33], [35] did not report the pharmacologic therapy. One trial [32], [34] performed a 3 month post-intervention follow up to assess the maintenance of intervention gains while the other trials only conducted testing immediately after the intervention. One trial [38] was excluded from the quantitative analysis due to a lack of important outcome data. The details of study characteristics are provided in Tables 1 and 2.

**Table 1:** Characteristics of the participants in the included studies.

Source	Location	Number of centers	Number of patients	Gender (M/F)	Age (years)	Disease severity (H & Y scale stage)	Disease duration (years)
Choi HJ 2013 ^[30]^	Seoul, Republic Korea	1	20	/	60.81±7.6/65.54±6.8	1.6±0.6/1.8±0.3	5.2±2.7/5.2±2.7
Nocera JR 2013 ^[32]^	Georgia, USA	1	21	11/10	66±11/65±7	2–3/2–3	8.08±5.42/6.83±1.83
Amano S 2013 (project 1) ^[33]^	Georgia, USA	1	21	14/7	64±13/68±7	2.3±0.4/2.2±0.4	7±7/12±7
Amano S 2013 (project 2) ^[33]^	Florida, USA	1	24	14/10	66±11/66±7	2.4±0.6/2.4±0.4	8±5/5±3
Li F 2012 ^[34]^ and Li F 2013 ^[32]^	Oregon, USA	4	195	122/73	68±9/69±8^a^/69±9^b^	2.5	8±9/8±9/6±5
Gladfelter BA 2011 ^[35]^	Indiana, USA	1	17	12/5	72±8.52	2.4±0.87	5.88±3.48
Zhu Y 2011 ^[36]^	Taipei and Shanghai, China	2	38	23/17	63.35±8.72/64.83±9.29	1–2	2.27±1.95/2.78±2.29
Li JX 2011 ^[37]^	Taipei, China	1	47	22/25	68.28±6.62/67.13±6.73	2.5–3	5.62±3.94/5.71±30.79
Hackney ME 2009 ^[38]^	St. Louis, USA	1	61	45/16	64.9±2.3/66.8±2.4^c^/68.2±1.4^d^/66.5±2.8^e^	2.0±0.1/2.0±0.2/2.1±0.1/2.2±0.2	8.7±1.3/9.2±1.4/6.9±1.3/5.9±1.0
Hackney ME 2008 ^[39]^	St. Louis, USA	1	26	21/5	64.9±8.3/62.6±10.2	2.0/2.0	8.7±4.7/5.5±3.3

**Notes**: Sources are stated as first author and publication year. Continuous data describing age, disease severity and disease duration are presented as mean ± SD or median or range. H & Y is for Hoehn and Yahr.

a Resistance exercise.

b Stretching.

c Waltz with Foxtrot.

d Tango.

e No intervention.

**Table 2:** Characteristics of the included studies.

Source	Intervention	Control	Outcomes
Choi HJ 2013 [^30^]	Tai Chi: 50 min/session, three times a week for 12 weeks with stable medication	Stable medication alone	UPDRS, OLS,TUG,TS,SMW, RTLS
Nocera JR 2013 [^31^]	Tai Chi: Short forms of Yang-style movements, 60 min/session, three times a week for 16 weeks with stable medication.	Stable medication alone	DSB-WMS, LVF, CVF, SCWT, Trail A-B, Tinetti's FE, PDQ-39
Amano S 2013 (project 1) [^33^]	Tai Chi: Eight Yang-style movements, 60 min/session, twice a week for 16 weeks	Qigong	Gait initiation, gait performance (cadence, gait velocity, step length, step duration, swing time, double-limb support time, gait asymmetry), UPDRS III.
Amano S 2013 (project 2) [^33^]	Tai Chi: Eight Yang-style movements, 60 min/session, three times a week for 16 weeks.	No intervention	
Li F 2012 [^34^] and Li F 2013 [^32^]	Tailored Tai Chi: Six movements integrated nto an eight-form routine, 60 min/session, twice a week for 24 weeks with stable medication.	a. Resistance training with stable medication. b. Stretching with stable medication	Postural stability (maximum excursion, directional control), gait performance (stride length, gait velocity), peak knee extension and knee flexion torque, FRT, TUG, UPDRS III, falls; short version of PDQ-39 (PDQ-8),VPS, UPDRS-ME, 50-foot speed walk test, continuing exercise measure
Gladfelter BA 2011 [^35^]	Tai Chi: Short forms of Yang-style movements, 60 min/session, once a week for 12 weeks.	Routine physical exercise	BBS, FRT, TUG, PDQ-39, falls, PPS.
Zhu Y 2011 [^36^]	Tai Chi: 24 movements of national standard, 30–45 min/session, twice a day, five times a week for 4 weeks, with Madopar	Walking exercise with Madopar	UPDRS III, BBS.
Li JX 2011 [^37^]	Tai Chi (24 movements of national standard, 30–45 minutes/session, twice a day, 5 times a week for 8 weeks) with Madopar	Walking exercise with Madopar	UPDRS III, BBS, PDQ-39SI.
Hackney ME 2009 [^38^]	Tai Chi: Short forms of Yang-style movements, 60 min/session, twice a week for 13 weeks with stable medication.	a. Waltz with Foxtrot. b. Tango. c. No intervention.All groups received stable medication	PDQ-39 and PDQ-39SI
Hackney ME 2008 [^39^]	Tai Chi (short forms of Yang-style, 60 minutes/session, once a week for 13 weeks) with stable medication.	Stable medication alone	UPDRS III, BBS,TUG, TS, OLS, gait (backward FAP, backward stride length, backward velocity, forward FAP, forward stride length, forward velocity), SMW, PPS.

**Notes**: UPDRS III, Unified Parkinson's Disease Rating Scale Part III; TUG, Timed Up and Go test; PDQ-39/8, Parkinson's Disease Questionnaire-39/8; PDQ-39SI, PDQ-39 Summary Index; BBS, Berg Balance Scale; FRT, Functional reach test; TS, Tandem stance test; OLS, One-leg stance test; FAP, Functional ambulation profile; SMW, Six minute walk; PPS, Post-program survey; DSB-WMS, Digit Span Backward Subtest from Wechsler Memory Scale; LVF, Letter Verbal Fluency; CVF, Category Verbal Fluency; SCWT, Stroop Color Word Test; Trail A–B, Trails A and B; Tinetti's FE, Tinetti's Falls Efficacy Scale; RTLS, reaction time upon light signal; VPS, Vitality Plus Scale; UPDRS-ME, Modified Unified Parkinson's Disease Rating Scale Motor Examination.

### 3. Risk of bias

**Figure 2:**
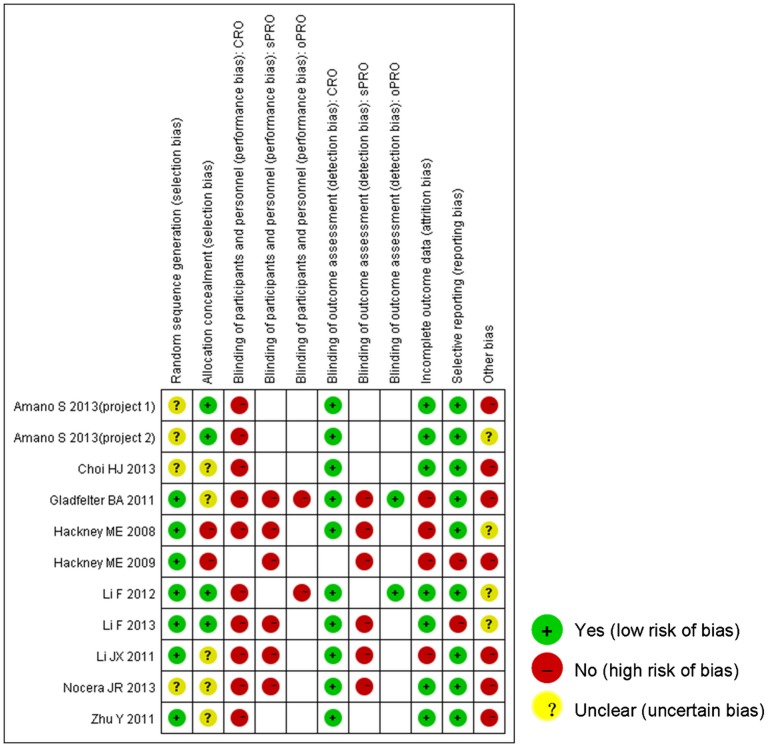
Risk of bias summary. The article [33] reported two independent trials that are labeled as project 1 and 2. Blank entries mean that the outcome was not reported. CRO, clinician-reported outcome; sPRO, subjective patient-reported outcome; oPRO, objective patient-reported outcome.

The protocol was registered and published for only one trial [32], [34]. For the other nine trials [30]–[31], [33], [35]–[39], the risk of bias was assessed according to the methods section of the published article. Six trials [32], [34]–[39] reported the method of randomization sequence generation, which included computer generation, randomization number table, coin toss and drawing of lots. Five trials [30], [31], [35]–[37] lacked detailed descriptions on whether allocation concealment was performed or not while two trials [38], [39] did not use allocation concealment judged by the articles and authors' responses. None of the trials blinded the participants or healthcare providers. All trials used independent outcome assessors who were unaware of intervention assignment, except patient-reported outcomes. All trials reported a low drop-out rate and provided a clear description of reasons for drop out, but AE might result in missing data in five trials [35]–[39]. Important outcome measurement data were not reported in two trials [32], [38], but the other trials demonstrated consistency of methods and results. Unreliable randomization was found in one trial [31], control group lacking validation occurred in four trials [33], [36]–[38] and extremely small sample size existed in two trials [30], [35]. The summary assessments of risk of bias are presented in Figure 2.

### 4. Data available for analysis

Of the ten included studies, only nine trials [30]–[37], [39] with 409 participants had data available for meta-analysis because one [38] did not report its single outcome data. Data from two articles [32], [34] reporting different outcomes of the same trial were synthesized as one trial in the meta-analysis. The number of patients practicing Tai Chi was divided up and 2 independent comparisons (labeled as comparison 1 and 2) were finally included into the data analysis for the trial with 3 groups [32], [34]. Standard mean difference (Std.MD) was presented in the effect of HRQOL and Gait for different scales or units were used. The results of falls, PPS and adverse events were presented with paragraph description rather than statistical synthesis due to the varied measuring methods. Publication bias was not detected because none of the groups included more than 10 studies.

### 5. Primary outcome

**Figure 3:**
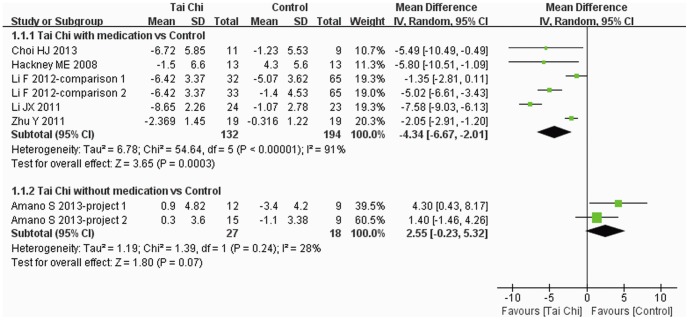
The effect of Tai Chi on UPDRS III score. Subgroup analysis was performed according to whether or not medications were included in the intervention. A random model was used to address the high heterogeneity. UPDRS III, Unified Parkinson's Disease Rating Scale Part III.

UPDRS III was reported in seven trials [30], [33], [34], [36], [37], [39] and subgroup analysis was performed. Reduction of total UPDRS III score was greater for Tai Chi plus medication than other exercise plus medication and medication alone (MD: −4.34, 95% CI: −6.67–−2.01). But there was no difference between Tai Chi without medication and no intervention and qigong alone (MD: 2.55, 95% CI: −0.23–5.32) (Figure. 3).

### 6. Secondary outcomes

#### 6.1. Balance and mobility function

**Figure 4:**
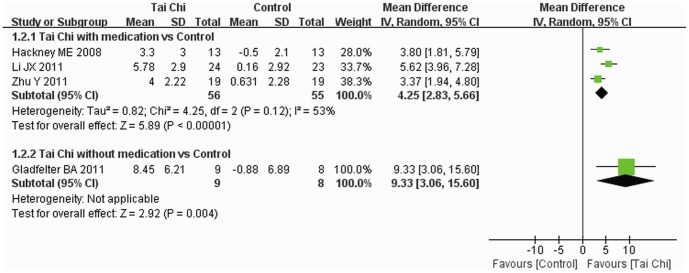
The effect of Tai Chi on BBS score. Subgroup analysis was performed according to whether or not medications were included in the intervention. A random model was used to address the high heterogeneity. BBS, Berg Balance Scale.

BBS was reported in four trials [35]–[37], [39] and subgroup analysis was performed. Increase in total BBS score was greater for Tai Chi plus medication than other exercise plus medication and with medication alone (MD: 4.25, 95% CI: 2.83–5.66), and the improvement was also greater for Tai Chi without medication than other exercise alone (MD: 9.33, 95% CI: 3.06–15.60) (Figure. 4).

**Figure 5:**
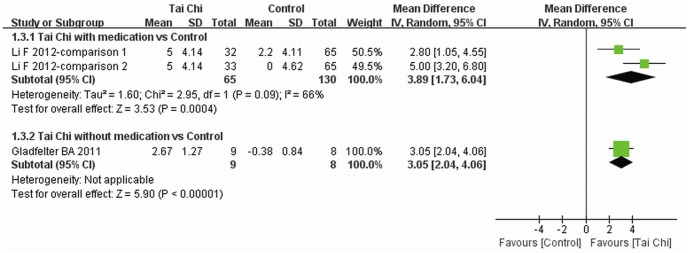
The effect of Tai Chi on FRT. Subgroup analysis was performed according to whether or not medications were included in the intervention. A random model was used to address the high heterogeneity. FRT, functional reach test.

FRT was reported in two trials [34], [35] and subgroup analysis was performed. Increase in maximal reach distance was greater for Tai Chi plus medication than other exercises plus medication (MD: 3.89, 95% CI: 1.73–6.04), and the improvement was also greater for Tai Chi without medication than with other exercise alone (MD: 3.05, 95% CI: 2.04–4.06) (Figure. 5).

**Figure 6:**
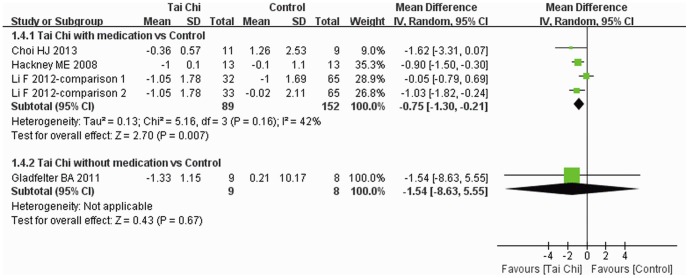
The effect of Tai Chi on TUG. Subgroup analysis was performed according to whether or not medications were included in the intervention. A random model was used to address the high heterogeneity. TUG, Timed Up and Go test.

TUG was reported in four trials [30], [34], [35], [39] and subgroup analysis was performed. Reduction in TUG time was greater for Tai Chi plus medication than other exercise plus medication and medication alone (MD: −0.75, 95% CI: −1.30–−0.21), but there was no significant difference between Tai Chi without medication and other exercise alone (MD: −1.54, 95% CI: −8.63–5.55) (Figure. 6).

#### 6.2. HRQOL

**Figure 7:**
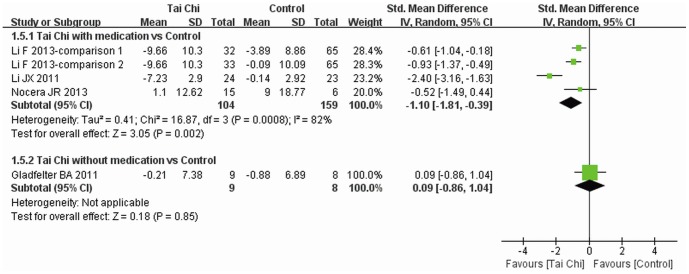
The effect of Tai Chi on HRQOL. Subgroup analysis was performed according to whether or not medications were included in the intervention. Standardized mean difference was calculated for PDQ-39 (short and long versions) and PDQ-39SI scores. HRQOL, health-related quality of life; PDQ-39, Parkinson's Disease Questionairre-39; PDQ-39SI, PDQ-39 Summary Index.

HRQOL was reported in four trials [31], [32], [35], [37] and subgroup analysis was performed. Three trials [31], [32], [35] reported the total score of PDQ-39 (long or short version) and one [37] introduced PDQ-39SI, so SMD was used for data analysis. Improvement of HRQOL was greater for Tai Chi plus medication than other exercise plus medication and medication alone (SMD: −1.10, 95% CI: −1.81–−0.39), but there was no significant difference between Tai Chi without medication and other exercise alone (SMD: −0.09, 95% CI: −0.86–1.04) (Figure. 7).

#### 6.3. Gait

**Figure 8:**
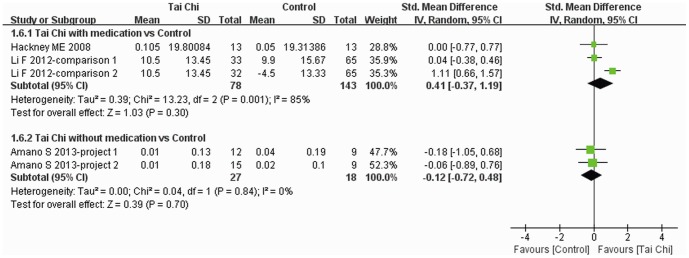
The effect of Tai Chi on gait velocity. Subgroup analysis was performed according to whether or not medications were included in the intervention. Standardized mean difference was used for different units of velocity.

Gait velocity was reported in four trials [33], [34], [39] and subgroup analysis was performed. Different units were introduced to report gait velocity in the four trials, therefore SMD was used for data analysis. There was no significant difference in the change in gait velocity between Tai Chi plus medication and other exercise plus medication and medication alone (SMD: 0.41, 95% CI: −0.37–1.19) or between Tai Chi without medication and no intervention and other exercise alone (SMD: −0.12, 95% CI: −0.72–0.48) (Figure. 8).

**Figure 9:**
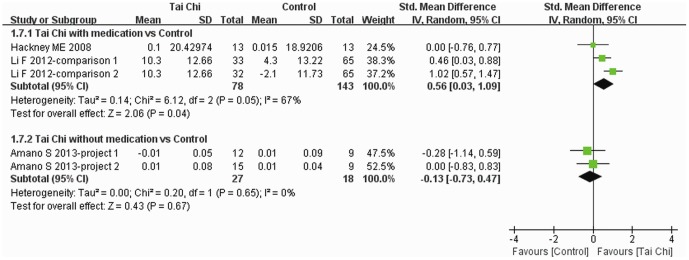
The effect of Tai Chi on stride length. Subgroup analysis was performed according to whether or not medications were included in the intervention. Standardized mean difference was used for different units of stride length.

Stride length was reported in four trials [33], [34], [39] and subgroup analysis was performed. Different units were introduced to report stride length in the four trials, so SMD was used for data analysis. The increase in stride length was greater for Tai Chi plus medication than other exercise plus medication and medication alone (SMD: 0.56, 95% CI: 0.03–1.09), but there was no difference between Tai Chi without medication and no intervention and other exercise alone (SMD: −0.13, 95% CI: −0.73–0.47) (Figure. 9).

#### 6.4. Falls

Falls data were obtained by patient self-report diaries in two trials [34], [35]. Patients were provided with a clear definition of falls before the intervention and required to record any falls they experienced. Patients [34] who received a 6 month Tai Chi intervention with stable medication had 67% fewer falls than patients who received a 6 month stretching intervention with medication (incidence-rate ratio: 0.33, 95% CI: 0.16–0.71), and marginally fewer falls than patients who received a 6 month resistance training intervention with medication (incidence-rate ratio: 0.47, 95% CI: 0.21–1.00). However, patients [35] who received a 12 week Tai Chi intervention without medication reported a similar rate of falls as patients who received no intervention.

#### 6.5. PPS

A PPS was conducted in two trials [35], [39] but different questionnaires were used to assess opinions on the experience of Tai Chi and the improvement in physical well-being. Patients [39] who received a 13 week Tai Chi intervention with medication enjoyed the class and somewhat agreed that the intervention improved balance, walking, co-ordination, endurance and mood, but did not agree that the intervention increased strength. Patients [35] who received a 12 week Tai Chi intervention supported and enjoyed the exercises.

#### 6.6. AE

Only one trial [32], [34] reported safety as a planned outcome. Six trials [34]–[39] reported AEs and four trials [30], [31], [33] did not report AE. None of the trails further defined the AE as adverse effects of Tai Chi. No serious AEs such as hospitalization, disability or death were noted, but minor AEs including pain, falls, dizziness and other symptoms were reported in the small population.

## Discussion

Tai Chi has been recommended as a regimen of life promotion [40] and falls reduction [41] in China and other countries. However, the efficacy of Tai Chi on PD is not well known. This is the first systematic review and meta-analysis to address the effect of Tai Chi in the management of PD and provides a new level of evidence for clinical professionals.

Our primary finding was that Tai Chi plus medication resulted in significantly greater benefit in terms of general motor symptoms, balance, mobility, and stride length, with few AE. Rigidity is one of the main manifestations of PD, leading to impairments in postural stability, balance and gait performance, and these symptoms do not respond well to either first-line or sub-optimal medications [42]. The movements of Tai Chi include weight shift, body rotation, slow strides and single-leg standing in different positions, requiring delicate joint control with muscle co-ordination [40]; therefore Tai Chi possibly trains postural stability and balance. This suggests that the combination of Tai Chi and medications may be optimal for PD patients who are partially insensitive to the pharmacologic treatment alone, especially if they have poor mobility and balance.

UPDRS III is most widely used for both clinical and research purposes to assess the motor function of PD patients [27] and we set it as our primary outcome in this systematic review. It has been previously reported that the minimal clinical relevant difference (MCRD) of UPDRS III is the improvement by 2.3–2.7 points [43] or by 5 points [44]. In our meta-analysis, a significant improvement of UPDRS III by 4.34 points in the group of Tai Chi plus medication was observed after pooled analysis, which is approaching the MCRD. This similarity means that the evidence of Tai Chi to improve motor symptoms of PD could be put in a real clinical situation.

The patients included in this review all had PD of mild-to-moderate severity, so the positive effect of Tai Chi cannot be extrapolated outside of this population. Tai Chi is one type of active physical exercise that can be used in place of passive rehabilitation strategies, but patients must have a basic ability to stand and move independently for certain duration. PD patients who are in the latter stages of the disease or in the “off” period of medication may not complete a Tai Chi intervention because of severe rigidity [45]. Therefore, the initiation of a Tai Chi program has to be carefully timed after the assessment of the disease severity and overall patient conditions.

Patient enjoyment and support for a therapy should be considered when deciding on a medical intervention [46]. We found a high satisfaction rate for Tai Chi intervention in the studies included in this review, which suggests that Tai Chi is feasible in clinical settings.

HRQOL reflects the overall influence of a disease on patients' physical mobility, daily activity, social functioning, psychological wellbeing and cognition [47], and is assessed from the point of view of the patient. Our research found that Tai Chi plus medications resulted in a greater improvement in HRQOL than other exercise plus medication and medication alone. This suggests that Tai Chi has an additional beneficial effect or better interaction with medication regarding PD-related health status, which is important for patients. However, knowledge of the assigned intervention may impact on patient-reported outcomes and the estimated effect has been observed to be more biased in trials with more subjective outcomes [48]. Based on the fact that it is not easy to blind participants practicing Tai Chi, it is therefore important to draw a conclusion in real situations considering both HRQOL reported by patients and other types of outcomes (such as objective outcomes and outcomes reported by caregivers or clinicians).

Falls are common and sometimes life threatening for PD patients [49]. A high score in the Freezing of Gait Questionnaire, older age, and the presence of falls in medical history are independent risk factors for falls [50]. We found that the reduction in falls was greater after a long-term (e.g. 6 months) Tai Chi program plus stable medications than other exercise plus medication, but that the reduction in falls after a short Tai Chi program without medication was not different from other exercise alone. However, the inconsistent results of Tai Chi in falls prevention were only obtained from two trials with small sample sizes, distinct designs and lack of comparative information. Therefore, the only conclusion we can draw is that the combination of Tai Chi with medications may be more effective for fall prevention, but further studies are needed on this topic.

However, performance of Tai Chi program without medication only had a beneficial effect on BBS and FRT, indicating that Tai Chi is not strong enough to combat all aspects of PD impairments.

### Limitations for the review

There are five main limitations to this review. First, the trials included had a small sample size and were conducted in only a few centers, which may bias the conclusions and further updates are needed to synthesize newly published eligible studies. Second, information recording the dosage and course of medications and evidence on whether Tai Chi reduces complications and side effects of long-term medications were not reported in any study, and these issues raise some uncertainty when recommending Tai Chi for PD patients. Third, long-term outcome of Tai Chi on PD was not investigated based on current evidence and further RCTs focusing on the effect of Tai Chi over a much longer period are needed for PD is a long-term disease [51]. Fourth, the logistics of balancing the benefits of Tai Chi with its economic cost and service availability were not analyzed in any study. Last, little evidence was found in this review as to whether the effects of Tai Chi are specific or non-specific, whether the promising effects are additive or synergistic with medication, and whether the effects can be attributed to a complex system rather than a single exercise [52]–[53].

### Difference from existing studies

Up to now, there are two systematic reviews trying to evaluate the effect of Tai Chi for PD [23, 54]. However, both of them only describe the outcomes rather than performing a meta-analysis to provide a pooled effect, which is not enough for new evidence.

## Conclusions

Tai Chi program performed with medication resulted in promising gains in mobility and balance and it was safe and popular among PD patients at an early stage. These results provide new evidence for PD management. More multi-center RCTs with a large sample size that carefully address blinding and prudently select outcomes are needed to confirm these results and to assess the feasibility of Tai Chi intervention for different medical situations. Research on the mechanism by which Tai Chi benefits PD patients is also required.

## Supporting Information

Checklist S1 **PRISMA Checklist.**
(DOCX)
